# Identifying Dysphagia and Demographic Associations in Older Adults Using Electronic Health Records: A National Longitudinal Observational Study in Wales (United Kingdom) 2008–2018

**DOI:** 10.1007/s00455-022-10425-5

**Published:** 2022-02-25

**Authors:** Joe Hollinghurst, David G. Smithard

**Affiliations:** 1 Swansea University, Singleton Park, Sketty, Swansea, SA2 8PP UK; 2 Queen Elizabeth Hospital, Lewisham and Greenwich NHS Trust, Stadium Road, Woolwich, London, SE18 4QH UK; 3 University of Greenwich, Avery Hill Campus, Bexley Rd, London, SE9 2PQ UK

**Keywords:** Frailty, Old age, Dysphagia, Prevalence, Epidemiology, Deprivation

## Abstract

Dysphagia is increasingly being recognised as a geriatric syndrome (giant). There is limited research on the prevalence of dysphagia using electronic health records. To investigate associations between dysphagia, as recorded in electronic health records and age, frailty using the electronic frailty index, gender and deprivation (Welsh index of multiple deprivation). A Cross-sectional longitudinal cohort study in over 400,000 older adults was undertaken (65 +) in Wales (United Kingdom) per year from 2008 to 2018. We used the secure anonymised information linkage databank to identify dysphagia diagnoses in primary and secondary care. We used chi-squared tests and multivariate logistic regression to investigate associations between dysphagia diagnosis and age, frailty (using the electronic Frailty index), gender and deprivation. Data indicated < 1% of individuals were recorded as having a dysphagia diagnosis per year. We found dysphagia to be statistically significantly associated with older age, more severe frailty and individuals from more deprived areas. Multivariate analyses indicated increased odds ratios [OR (95% confidence intervals)] for a dysphagia diagnosis with increased age [reference 65–74: aged 75–84 OR 1.09 (1.07, 1.12), 85 + OR 1.23 (1.20, 1.27)], frailty (reference fit: mild frailty 2.45 (2.38, 2.53), moderate frailty 4.64 (4.49, 4.79) and severe frailty 7.87 (7.55, 8.21)] and individuals from most deprived areas [reference 5. Least deprived, 1. Most deprived: 1.10 (1.06, 1.14)]. The study has identified that prevalence of diagnosed dysphagia is lower than previously reported. This study has confirmed the association of dysphagia with increasing age and frailty. A previously unreported association with deprivation has been identified. Deprivation is a multifactorial problem that is known to affect health outcomes, and the association with dysphagia should not be a surprise. Research in to this relationship is indicated.

**Supplementary Information:**

The online version contains supplementary material available at 10.1007/s00455-022-10425-5.

## Introduction

### Background

Older people are a becoming a larger percentage of the world’s population, with 15% of the UK population aged 65 years and over [1, 2]. In the UK demography, the 85 + age group (very old) is the fastest growing cohort and is estimated to double to 3.2 million by mid-2041 and treble by 2066 (5.1 million; 7% of the UK population) [3–6]. More concerning is that over the last 15 years, there has been an 85% increase in the number of centenarians, which is predicted to reach 21,000 by 2030 [6].

Swallowing is a complex interplay of the cerebral cortex and brainstem, several cranial nerves and multiple muscles in the head and neck [7]. The main purpose of swallowing is to transfer food safely from the mouth to the stomach whilst protecting the airway. There are three sequential phases of swallowing: oral, pharyngeal and oesophageal. The pharyngeal swallow is semi-automatic and occurs in a coordinated sequence, the timings of which will be modulated by the cortex depending on the characteristics of the bolus/food swallowed [8]. Dysfunction (secondary to localised or generalised disease processes) in one or more of the phases may result in dysphagia (difficulty swallowing) [9, 10]. Dysphagia is defined as a subjective sensation of difficulty or abnormality of swallowing [11]. This definition encompasses oropharyngeal dysphagia, oesophageal dysphagia and reflux disease.

Changes to eating and swallowing may occur with age, with some individuals accepting this as a normal part of ageing, slowly adapting the quantity and texture of food eaten and the time taken to eat, often referred to as presbyphagia [11, 12]. Physiological changes to the swallow with increasing age may vary with gender and between individuals. In general, it has been found that there is reduced hyoid excursion, laryngeal elevation and anterior rotation in individuals with dysphagia. Transit times through the pharynx increase, and UES opening is prolonged [13]. When does a change in swallow pattern become pathological? Humbert and Robbins [14] cautioned against labelling a “normal yet safe swallow” as being evidence of dysphagia. McCullough et al. comment that laryngeal penetration was more frequent and deeper in the older age group, but that clearance was the usual outcome [15]. Presbyphagia [11] is defined as “characteristic changes in the mechanism of oropharyngeal/oesophageal swallowing of otherwise healthy older adults” which may manifest as fatigue [16] and slowness of eating rather than overt dysphagia. The identified physiological changes with age [17, 18] may be directly or indirectly secondary to frailty and pre-existing comorbidities, rather than age specific.

There have been many prevalence/epidemiological studies of dysphagia in community living older people [15, 19–26]. These surveys have been completed over three decades and have used several different methodologies including postal surveys, cold-calling telephone calls and clinic waiting room approaches. The studies have also used variable definitions of “old” and of what constitutes dysphagia [27], which makes comparisons difficult.

Determining/understanding the prevalence of dysphagia in the community is complex. Studies are referenced to a particular country, set environment or disease state, rather than an unselected population. Often the definition of dysphagia is vague or not stated. The true prevalence of dysphagia in the community is uncertain, with a wide range from 2.5 to 72% [17, 25, 27–32] reported. Adkins et al. reported a prevalence of 16.1% (4998/31129) across all age groups [20], whereas Battacharyya reported a 4% prevalence from a cohort of 30,000 in a household survey [33]. Kertscher et al. using EAT-10 as part of a telephone survey, found that 21.9% of those > 76 years scored ≥ 3, indicating problems swallowing [21]. A study by Andersen et al. found that 26% of participants reported difficulty taking tablets [34]. In two studies of community living older (> 65 years) adults, at different time points [35, 36], 33.7% of those in Korea were found to have symptoms of dysphagia, whilst the prevalence was only 13.8% in Japan. Patel et al. reported that 3.1% of all adult admissions to hospital had a diagnosis of dysphagia [37].

Frailty is a modern geriatric syndrome (giant) [38], but identifying frailty can be difficult due to the lack of a universally accepted definition [18]. Frailty has been defined by some as “a biologic disorder affecting many activities of daily living characterised by diminishing physiological reserves [39, 40] which may be due to an accumulation of multiple system deficits” [41], and as a “state of reduced strength and physiological malfunctioning that increases a person’s susceptibility to increased dependency, vulnerability and even death” [42, 43]. It is generally accepted that the prevalence of frailty increases with age, with approximately 25–50% of people over the age of 85 years being frail [44]. In hospital populations, studies have shown that frailty and dysphagia frequently co-exist [14, 45, 46] but there remains a debate as to whether there is a distinct entity of frailty dysphagia [14]. Recent papers [38, 47] define oral frailty—but not frailty dysphagia—as a decrease in oral functioning together with a decline in cognitive and physical function but fail to fully develop the discussion. Tanaka et al. noted that poor oral status in ≥ 1 oral domain was a predictor for physical frailty, suggesting association rather than a distinct entity of oral frailty. However, it is to be expected that dysphagia co-exists with frailty due to the suprahyoid muscle complex and the masseter muscle being skeletal in nature [48].

Frail older adults admitted to hospital have a prevalence of dysphagia of 55% [14, 45]. Cha et al. report that sarcopenia and dysphagia are concurrent in 6% of community dwelling older adults (> 65 years) [49]. Hansen et al. found that at a follow-up assessment of 56 weeks after discharge from hospital, 25% of older adults had dysphagia and that there was a significant relationship at the *p* < 0.05 level with sarcopenia, low physical performance and malnutrition [50]. These changes, combined with changes to food preferences, have the potential to result in under-nutrition [18]. These inter-relationships contribute to a “failure to thrive” and have the potential to accelerate people towards a terminal physical decline ultimately resulting in death.

Nursing home populations have a greater prevalence due to many residents having a diagnosis of dementia, stroke or Parkinson’s disease alone, or in combination, with frailty [51, 52]. In a systematic review by Takizawa et al., dysphagia was diagnosed in 8.1–80% of stroke patients, 27–30% of brain injury patients, 11–81% of Parkinson disease patients and 91.7% of older persons with community-acquired pneumonia due to impaired swallowing and breathing mechanisms [53].

In the clinical situation, despite clinical staff being aware that dysphagia is a complication of many aetiological mechanisms, including mechanical, neurological and psychological [54], it is nevertheless often under-diagnosed and under-treated [55]; as a consequence, there is an increased risk of malnutrition, dehydration, pneumonia [56] and mortality [57] in the relevant populations.

### Rationale for the Study

Studies may over or under report the prevalence of dysphagia in the community depending on the research approach taken. Bias may be introduced by both the survey and the researcher. Those studies of dysphagia focussing on older people have tended to be small [17, 26], and larger studies have relied on census data [24, 33]. No studies have examined the medical data held in electronic health records (EHRs) that have been collated as part of day-to-day clinical care and explored the relationship with and age, gender, frailty and deprivation.

The use of existing anonymised routinely collected longitudinal data can help provide rapid access to large-scale data for studies and provide robust evidence for commissioning decisions and policy [58, 59]. In this study, we utilise the Secure Anonymised Information Linkage (SAIL) Databank [60–62] to investigate the recording of dysphagia in EHRs in older adults in Wales over time. We also include linked data to explore associations between dysphagia and age, gender, frailty and deprivation.

## Methods

### Study Design

We used anonymised EHRs and administrative data from the SAIL Databank to create a longitudinal cross-sectional cohort study.

For the purposes of this study, we have accepted dysphagia as any entry in to the EHR that documents dysphagia or swallowing problem/difficulty.

### Data Sources

Our cohorts were created using data held within the SAIL Databank [60–62]. The SAIL Databank contains longitudinal anonymised administrative and healthcare records for the population of Wales. The anonymisation is performed by a trusted third party, the National Health Service Wales Informatics Service. The SAIL Databank has a unique individual anonymised person identifier known as an anonymous linking field and a unique address anonymised identifier known as a residential anonymous linking field [63] that are used to link between data sources at individual and residential levels, respectively. Individual linking fields, nested within residential codes, are contained in the anonymised version of the Welsh Demographic Service Dataset (WDSD), replacing the identifiable names and addresses of people registered with a free-to-use General Practitioner service.

The WDSD contains demographic information including week of birth, gender, date of death and lower-layer super output area (LSOA). Each LSOA in Wales has been ranked from most deprived to least deprived according to the Welsh Index of Multiple Deprivation (WIMD), an area-based measure of socio-economic status [64]. We used the 2011 version of LSOA linked to the 2014 version of the WIMD and used deprivation quintiles in the analyses. To detect dysphagia in older adults, we used both the Welsh Longitudinal General Practice (WLGP) dataset and the Patient Episode Database for Wales (PEDW). The WLGP contains general practice data for approximately 80% of the population of Wales, and we used Read codes version 2 (CTV2) to identify dysphagia. The PEDW dataset contains all hospital admission records in Wales, and we identified individuals with dysphagia in these records using the international classification of disease version 10 (ICD-10) code for dysphagia.

### Setting and Participants

We included all individuals aged 65 + living in Wales with a valid address who were registered with a general practice contributing data to the SAIL databank. We created 11 cohorts independently, one for each year from 2008 to 2018. Dysphagia was identified in the previous year from the cohort index dates to mitigate the need for censoring due to death or migration. For example, the cohort created for 2008 had index date 1st January 2008, and dysphagia was identified from 1st January 2007 to 31st December 2007.

### Variables

Our outcome of interest was dysphagia identified in either general practice or hospital records. We used CTV2 Read codes to identify dysphagia in general practice records and ICD-10 codes to identify dysphagia in hospital records. We created binary indicators for dysphagia for each person, each year if dysphagia was identified in either general practice, hospital records, or both in the year prior to the cohort index date. No restriction was placed on the number of codes searched in either general practice or hospital records. The cohort index dates were treated as categorical and included as the year of the cohort index date (2008 to 2018). Age was calculated on the cohort index date and was categorised in to three groups: 65–74, 75–84, 85+. Gender (male/female) was treated as categorical variable. Frailty was calculated using the electronic Frailty index (eFI). The eFI is based on an internationally established cumulative deficit model and assigns a frailty score to an individual calculated using 36 variables from primary care GP data, including symptoms, signs, diseases, disabilities and abnormal laboratory values, referred to as deficits [65]. The eFI score is the number of deficits present, expressed as an equally weighted proportion of the total. An individual with a single deficit would be assigned an eFI of 1/36 (0.03); another with nine deficits would be assigned an eFI of 9/36 (0.25). The eFI score is used to categorise individuals as fit (eFI value of 0–0.12), mild (> 0.12–0.24), moderate (> 0.24–0.36), or severely frail (> 0.36) [65]. We calculated the eFI retrospectively in the SAIL databank on the cohort index dates using 10 years of previous GP data for each individual each year [66]. This meant that for someone in the cohort for the 1st January 2010, the eFI was calculated using data from 1st January 2000 to 1st January 2010. The WIMD quintiles were included as categorical variables ranging from 1—Most Deprived, to 5—Least Deprived.

### Statistical Methods

We produced descriptive statistics for our cohorts and stratified these by individuals recorded as having dysphagia and those not. We computed chi-squared statistics with a significance level of 0.05 for each cohort year to investigate differences in age, frailty, gender and WIMD between individuals with and without a dysphagia diagnosis. We calculated multilevel logistic regression models to investigate associations between dysphagia recorded in the previous year and the demographic information. The multilevel logistic regression models included the cohort year as a random effect. Sensitivity analyses included independent multilevel logistic regression models for dysphagia diagnoses in primary care (general practice) and secondary care (hospital) records. Statistical analyses were performed using R version 4.0.0 [67] with R2MLwiN [68].

## Results

### Study Size

We developed our cohorts by limiting to only individuals aged 65 + on the index dates, this was further limited by restricting the cohort to individuals registered with a general practice submitting data to the SAIL databank. Finally, we restricted the cohorts to individuals with a valid residential address so we were able to determine the WIMD for each individual. The remaining number of people after these restrictions is recorded in the supplementary material, Table S1.

### Recording of Dysphagia and Differences in Demographic Information

The number of people diagnosed with dysphagia varied with each year. We further stratified the diagnoses by Read code and ICD-10 code. The numbers of codes are recorded in the supplementary material, Table S2. The results suggest an increase in the recording of dysphagia over time and an increase in the number of the types of codes used.

**Fig. 1:**
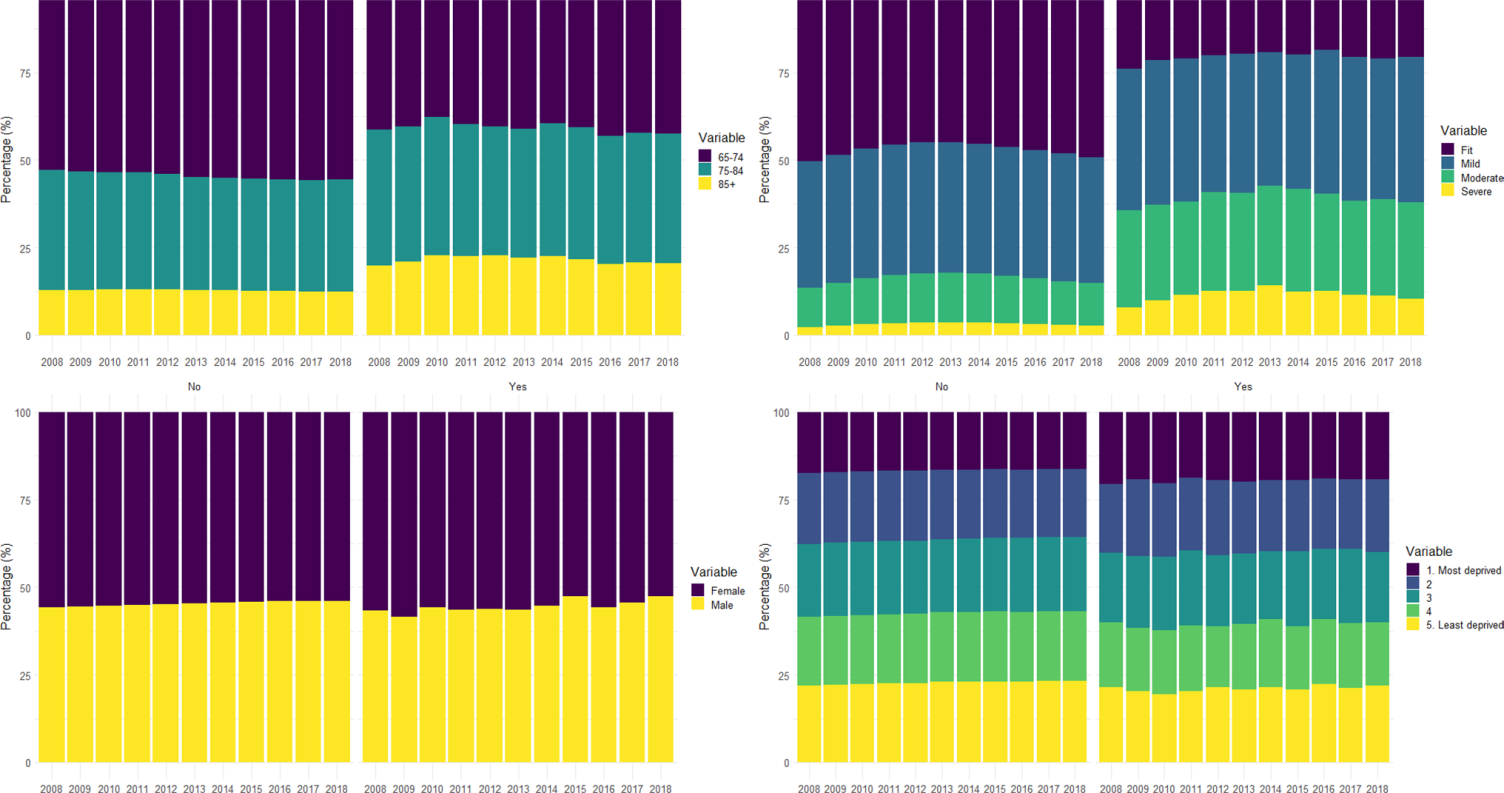
Demographic differences between individuals identified with dysphagia in hospital or general practice records in the year before cohort entry: 2008 and 2018

The demographic information over time for the cohorts is recorded in Table 1, with stratified breakdowns for people without a dysphagia diagnosis recorded in Table 2 and those with a dysphagia diagnosis recorded in Table 3. The change in demographic information over time is also displayed in Fig. 1. Tables 2, 3 and Fig. 1 suggest differences in age, frailty (eFI) and WIMD between those with and without a dysphagia diagnosis, but no significant difference in gender. Table S3 includes the p values for chi-squared tests for differences in proportions for age, frailty (eFI), gender and WIMD. With the exception of gender, all tests showed significant differences in distributions for individuals with a dysphagia diagnosis compared to those without in the majority of the years tested (2008, 2010–2012, 2014–2015, and 2017–2018).

**Table 1:** Overall cohort characteristics—Individuals with a general practice registration on the cohort index date

Year	2008	2009	2010	2011	2012	2013	2014	2015	2016	2017	2018
*Individuals*											
Count (*N*)	400,921	407,983	417,824	424,317	437,693	450,160	464,122	475,882	486,620	495,186	502,791
*Dysphagia identified*											
All prior records	3.6%	3.8%	4.0%	4.3%	4.5%	4.7%	4.9%	5.1%	5.4%	5.6%	5.8%
Previous year	0.6%	0.6%	0.6%	0.6%	0.6%	0.7%	0.7%	0.7%	0.8%	0.7%	0.8%
General practice—all prior records	2.6%	2.8%	2.9%	3.1%	3.3%	3.4%	3.5%	3.7%	3.8%	3.9%	4.0%
General practice—previous year	0.4%	0.4%	0.4%	0.4%	0.4%	0.4%	0.4%	0.4%	0.4%	0.4%	0.4%
Hospital—all prior records	1.4%	1.5%	1.6%	1.8%	1.9%	2.0%	2.1%	2.2%	2.4%	2.6%	2.8%
Hospital—previous year	0.3%	0.3%	0.3%	0.3%	0.3%	0.3%	0.3%	0.4%	0.4%	0.4%	0.4%
*Age*											
65–74	52.8%	53.1%	53.4%	53.5%	54.0%	54.7%	55.0%	55.2%	55.6%	55.7%	55.4%
75–84	34.4%	33.9%	33.5%	33.3%	32.8%	32.3%	32.1%	32.0%	31.8%	31.7%	32.1%
85+	12.9%	13.0%	13.1%	13.2%	13.2%	13.0%	12.9%	12.8%	12.6%	12.5%	12.5%
*Frailty (eFI)*											
Fit	50.2%	48.3%	46.6%	45.4%	44.8%	44.9%	45.3%	46.2%	47.0%	48.0%	48.9%
Mild	36.2%	36.6%	36.9%	37.3%	37.3%	37.2%	37.0%	36.8%	36.7%	36.4%	36.1%
Moderate	11.4%	12.4%	13.3%	13.9%	14.2%	14.2%	14.0%	13.5%	13.1%	12.6%	12.2%
Severe	2.3%	2.7%	3.1%	3.4%	3.7%	3.8%	3.7%	3.5%	3.2%	3.0%	2.8%
*Gender*											
Female	55.8%	55.5%	55.3%	55.1%	54.9%	54.7%	54.5%	54.3%	54.1%	54.0%	53.8%
Male	44.2%	44.5%	44.7%	44.9%	45.1%	45.3%	45.5%	45.7%	45.9%	46.0%	46.2%
*WIMD 2014*											
1. Most deprived	17.3%	17.1%	16.9%	16.8%	16.7%	16.6%	16.4%	16.3%	16.5%	16.3%	16.2%
2	20.5%	20.3%	20.3%	20.1%	20.1%	19.8%	19.7%	19.6%	19.5%	19.5%	19.5%
3	20.8%	20.8%	20.9%	20.9%	20.9%	20.9%	21.0%	21.2%	21.2%	21.2%	21.2%
4	19.4%	19.6%	19.6%	19.6%	19.7%	19.8%	19.9%	19.9%	19.8%	19.8%	19.8%
5. Least deprived	22.0%	22.2%	22.4%	22.6%	22.6%	22.9%	22.9%	23.0%	23.1%	23.2%	23.3%

**Table 2:** No dysphagia identified in either hospital or general practice records in 1 year prior to cohort index date

Year	2008	2009	2010	2011	2012	2013	2014	2015	2016	2017	2018
*Individuals*											
*N*	398,409	405,496	415,266	421,588	434,882	447,104	460,896	472,593	482,893	491,508	498,970
*Age*											
65–74	52.8%	53.2%	53.5%	53.6%	54.1%	54.8%	55.2%	55.3%	55.7%	55.8%	55.5%
75–84	34.3%	33.9%	33.5%	33.3%	32.8%	32.3%	32.1%	32.0%	31.8%	31.7%	32.1%
85+	12.8%	12.9%	13.0%	13.1%	13.2%	12.9%	12.8%	12.7%	12.6%	12.5%	12.4%
*Frailty (eFI)*											
Fit	50.3%	48.4%	46.8%	45.6%	45.0%	45.0%	45.5%	46.4%	47.2%	48.2%	49.1%
Mild	36.1%	36.6%	36.9%	37.3%	37.3%	37.2%	37.0%	36.8%	36.6%	36.4%	36.1%
Moderate	11.3%	12.3%	13.3%	13.8%	14.1%	14.1%	13.9%	13.4%	13.0%	12.5%	12.0%
Severe	2.2%	2.6%	3.1%	3.4%	3.6%	3.7%	3.6%	3.4%	3.2%	2.9%	2.8%
*Gender*											
Female	55.8%	55.5%	55.3%	55.1%	54.9%	54.6%	54.5%	54.3%	54.1%	54.0%	53.9%
Male	44.2%	44.5%	44.7%	44.9%	45.1%	45.4%	45.5%	45.7%	45.9%	46.0%	46.1%
*WIMD 2014*											
1. Most deprived	17.3%	17.1%	16.8%	16.8%	16.7%	16.5%	16.4%	16.3%	16.4%	16.3%	16.2%
2	20.5%	20.3%	20.3%	20.1%	20.1%	19.8%	19.7%	19.6%	19.5%	19.5%	19.5%
3	20.8%	20.8%	20.9%	20.9%	20.9%	20.9%	21.0%	21.2%	21.2%	21.2%	21.2%
4	19.5%	19.6%	19.6%	19.6%	19.7%	19.8%	19.9%	19.9%	19.8%	19.8%	19.8%
5. Least deprived	22.0%	22.2%	22.4%	22.6%	22.7%	22.9%	23.0%	23.1%	23.1%	23.2%	23.3%

**Table 3:** Dysphagia identified in either hospital or general practice records 1 year prior to cohort index date

Year	2008	2009	2010	2011	2012	2013	2014	2015	2016	2017	2018
*Individuals*											
*N*	2,512	2,487	2,558	2,729	2,811	3,056	3,226	3,289	3,727	3,678	3,821
*Age*											
65–74	41.4%	40.5%	37.6%	39.7%	40.4%	41.2%	39.6%	40.6%	43.1%	42.3%	42.3%
75–84	38.7%	38.4%	39.7%	37.7%	36.9%	36.7%	37.8%	37.8%	36.7%	37.0%	37.2%
85+	19.9%	21.0%	22.7%	22.6%	22.7%	22.1%	22.6%	21.6%	20.3%	20.8%	20.4%
*Frailty (eFI)*											
Fit	24.0%	21.5%	21.0%	20.1%	19.6%	19.3%	19.9%	18.5%	20.6%	21.0%	20.5%
Mild	40.4%	41.2%	40.9%	39.0%	39.8%	38.0%	38.3%	41.2%	41.1%	40.1%	41.5%
Moderate	27.6%	27.5%	26.6%	28.4%	28.0%	28.5%	29.4%	27.6%	26.8%	27.6%	27.5%
Severe	8.0%	9.8%	11.4%	12.5%	12.5%	14.2%	12.4%	12.7%	11.5%	11.3%	10.4%
*Gender*											
Female	56.7%	58.5%	55.8%	56.5%	56.2%	56.4%	55.4%	52.6%	55.9%	54.4%	52.6%
Male	43.3%	41.5%	44.2%	43.5%	43.8%	43.6%	44.6%	47.4%	44.1%	45.6%	47.4%
*WIMD 2014*											
1. Most deprived	20.5%	19.1%	20.4%	18.8%	19.4%	19.9%	19.5%	19.4%	19.0%	19.3%	19.1%
2	19.7%	22.0%	21.1%	20.7%	21.4%	20.4%	20.3%	20.4%	20.1%	19.8%	20.9%
3	19.8%	20.6%	20.9%	21.4%	20.5%	20.2%	19.3%	21.5%	20.0%	21.3%	20.0%
4	18.6%	18.0%	18.2%	18.8%	17.3%	18.8%	19.4%	17.9%	18.5%	18.5%	18.1%
5. Least deprived	21.3%	20.3%	19.5%	20.3%	21.5%	20.7%	21.5%	20.8%	22.4%	21.1%	21.9%

### Multilevel Logistic Regression Results

The regression results are displayed in Table 4. Multivariate analyses indicated increased odds ratios [OR (95% confidence intervals)] for a dysphagia diagnosis for increased age [reference 65–74: aged 75–84 OR 1.09 (1.07, 1.12), 85 + OR 1.23 (1.20, 1.27)], frailty [reference fit: mild frailty 2.45 (2.38, 2.53), moderate frailty 4.64 (4.49, 4.79) and severe frailty 7.87 (7.55, 8.21)], and increased ORs for the most deprived areas [reference 5. Least deprived, 1. Most deprived: 1.10 (1.06, 1.14)]. In the univariate analysis, males had a decreased odds of dysphagia diagnosis [OR 0.97 (0.95, 0.99)] but increased odds in the multivariate analysis [OR 1.14 (1.11, 1.16)]. The null model has a small, but non-zero, variance at the cohort year level, which suggests that there is variance between the cohort years.

**Table 4:** Univariate and multivariate multilevel logistic regression model with fixed effects for age, frailty (electronic frailty index), gender and welsh index of multiple deprivation

Odds ratios (95% confidence interval)	Null	Age	Frailty	Gender	WIMD	Multivariate
*Age (reference: 65–74)*						
75–84	–	1.541 (1.504, 1.579)	–	–	–	1.093 (1.066, 1.120)
85+	–	2.240 (2.177, 2.305)	–	–	–	1.234 (1.196, 1.272)
*Frailty (electronic frailty index, reference: fit)*						
Mild	–	–	2.515 (2.443, 2.589)	–	–	2.453 (2.382, 2.527)
Moderate	–	–	4.897 (4.747, 5.053)	–	–	4.637 (4.487, 4.793)
Severe	–	–	8.478 (8.149, 8.821)	–	–	7.872 (7.548, 8.210)
*Gender (reference: female)*						
Male	–	–	–	0.971 (0.951, 0.992)	–	1.135 (1.111, 1.160)
*Welsh index of multiple deprivation 2014 (Reference: 5. least deprived)*						
1. Most deprived	–	–	–	–	1.269 (1.227,1.312)	1.100 (1.063, 1.138)
2	–	–	–	–	1.121 (1.085, 1.159)	1.010 (0.977, 1.044)
3	–	–	–	–	1.053 (1.019, 1.089)	0.973 (0.941, 1.006)
4	–	–	–	–	1.005 (0.972, 1.040)	0.955 (0.923, 0.989)
Intercept	0.007 (0.007, 0.007)	0.005 (0.005, 0.005)	0.003 (0.003, 0.003)	0.007 (0.007, 0.007)	0.006 (0.006, 0.007)	0.003 (0.003, 0.003)
*Random effects*						
Level 2 variance (annum)	0.006 (0.001, 0.012)	0.007 (0.001, 0.013)	0.008 (0.001, 0.014)	0.006 (0.001, 0.012)	0.006 (0.001, 0.012)	0.007 (0.001, 0.014)
–	–	–	–	–	–	–
Observations	4,963,499	4,963,499	4,963,499	4,963,499	4,963,499	4,963,499
Level 2 groups (years)	11	11	11	11	11	11

The cohort year was included as a random effect

### Sensitivity Analyses

The independent multilevel logistic regression results for the dysphagia diagnoses identified in primary and secondary care are displayed in the supplementary material, Tables S4 and S5, respectively. Results were consistent with the combined analysis but showed an increased odds of a recording of a dysphagia diagnosis in secondary care for those in both the most and second most deprived areas compared to the least deprived with ORs of 1.22 (1.17, 1.28) and 1.08 (1.03, 1.13), respectively.

## Discussion

This is the largest longitudinal study we are aware of to report on the recording of dysphagia using primary and secondary care records in a large cohort of older adults (65 + years). The research literature reports that older adults living in the community have a prevalence of dysphagia up to 72% [15, 17, 28, 69]. This study, in comparison, reports a markedly lower prevalence rate, with dysphagia reported at any time prior to the cohort survey date increasing from 3.6 to 5.8% between 2008 and 2018. The percentage of individuals with a diagnosis of dysphagia recorded one year prior to the cohort survey date was consistent over the decade, ranging from 0.6 to 0.8% in all those aged 65 + years. The large difference in the percentage of people with identified with dysphagia between our cohort and that reported in other studies raises questions as to what the true prevalence is. Interestingly, our results have shown a higher recording of dysphagia diagnosis in primary care EHR rather than hospital care records. This runs contrary to previous data [14, 45] but may be due to hospital data not relating solely to acute admissions, or the GP taking a more holistic approach, particularly with multiple comorbidities, including frailty who may be at greatest risk of developing dysphagia.

The results of our study confirm associations between age, frailty and identified dysphagia. Dysphagia is a complication of many other medical problems [28, 49], and the literature using hospital-based studies has noted an association between multimorbidity and dysphagia [14, 45, 70]. Our study extends this and reports the increased likelihood of identified dysphagia being present with differing levels of frailty severity identified using primary care records. Compared to individuals characterised as fit, we found increased ORs of 2.45 (2.38, 2.53), 4.63 (4.49, 4.79) and 7.87 (7.55, 8.21) for mild, moderate and severe frailty, respectively. As expected, we also found the likelihood of a dysphagia diagnosis increases with age. Compared to those aged 65–74, we noted increased ORs of 1.10 (1.07, 1.12) and 1.23 (1.20, 1.27) for those aged 75–84 and 85 +,  respectively. Although these findings are not novel, we have used a large dataset to provide robust evidence that frail older adults are particularly vulnerable and at a high risk of having, or potentially developing, dysphagia.

Our study has suggested an association between identified dysphagia and deprivation, with people living in the most deprived areas compared to the least deprived areas having an increased OR of 1.10 (1.06, 1.14). There are no documented studies we are aware of demonstrating this association. Deprivation is defined as a lack of access to basic needs such as food, shelter, clothing, health and education. Lower socio-economic class is associated with an increase in smoking, risky drinking habits, lack of exercise, poor diet [71] and often reduced self-care and increased risk of hospitalisations [72, 73], all synonymous with deprivation. A consequence of deprivation is the risk of malnutrition, illness, mortality, frailty and an increase in complex multimorbidity [74]. The increase in social deprivation, age and frailty (including oral frailty) manifests itself in the most vulnerable [14] in the form of an old age quartet (dysphagia, frailty, malnutrition and sarcopenia); with this subset of the population likely to have a particularly high prevalence of dysphagia. This is supported by our finding that the documentation of dysphagia in secondary care is more common [OR 1.100 (1.063, 1.138)] in people from deprived areas.

Our study has analysed data recorded as part of health and social care interaction, as opposed to using a survey. Studies show that many people do not report their swallowing difficulties to a health care professional. For example, Chen et al. found that 24% of those with swallowing problems did not report them to their family or doctor [75]. Wilkins et al. found that 2% of 947 people ≥ 18 years of age reported dysphagia at least several times a month, but 46% had not reported the problem to their GP [24]. Similarly, Adkins et al. reported that 2445 of 4998 (49%) survey participants had not consulted a health care practitioner [20]. In the USA, Bhattacharyya et al. estimated that 9.4 million adults had had dysphagia in the previous year [33]. This was extrapolated from a cohort of 1554 (4%) of the sample reporting dysphagia, where 22.7% (353) had seen a health care professional, 36.9% (130) had received a confirmed diagnosis and 56.5% reported that their swallow was a moderate/big/very big problem. It is possible that the failure to report swallowing problems, compounded with the failure to enquire about/screen for swallowing problems, accounts for some of the discrepancy in the prevalence between our study and previously reported data.

As all studies have asked a slightly different question regarding swallowing, swallowing problems or dysphagia, and some are more detailed than others, each study provides an independent, rather than complementary, part of the jigsaw. When considering studies that have used surveys and questionnaires, it should be noted that survey responses are influenced by the ability to understand the question, the ability to recall relevant information [76], the willingness of the participant to show normative behaviour and the willingness to please [77]. Face-to-face interviews are reliant on the interaction between the interviewer and the participant, including the behaviour of the interviewer [78], and in both telephone/postal and face-to-face interviews, the length of the survey may provoke a more negative response [79].

In summary, there are two possible interpretations for the differences in reported prevalence of dysphagia diagnoses in the literature and diagnosed dysphagia in our study: first, there are people in the community with swallowing difficulties who have not been identified and are not receiving support from either their GP, speech and language therapist, or dietitian; or second, the community prevalence of dysphagia is not as great as previously reported.

### Limitations

We included only individuals with a confirmed general practice registration on the cohort index date. We were unable to include 100% coverage of the Welsh population due to some general practices not contributing data to the SAIL databank. As with many data linkage studies, we did not have perfect matching between primary care and administrative records which meant some individuals were lost when including demographic information. In addition, we acknowledge the limitations of a diagnosis of dysphagia being recorded in electronic health records as it may not be a primary diagnosis, there may be errors in coding and in common with many studies, there is no clear definition of dysphagia.

The information collected and documented in medical records is limited by the need for specific codes which may not be sensitive enough to accurately represent medical problems. Health care professionals do not routinely ask or screen for swallowing [55] even if there are well-documented neurological disease such as stroke, dementia or Parkinson’s disease.

There is no accepted, unifying definition of dysphagia, and this study has used a series of read codes and ICD-10 codes to capture swallowing problems/dysphagia. Some of these codes may not directly relate to oropharyngeal dysphagia.

### Strengths

We performed a large longitudinal data linkage study of over 400,000 individuals per year. This adds significant and novel evidence for the levels of recording of dysphagia in older adults. We were able to link primary and secondary care data together with administrative data to gain insights to the associations between dysphagia and the included demographics.

## Conclusion

The study has identified a lower prevalence of diagnosed dysphagia than previously reported. The results have confirmed the association of identified dysphagia with increasing age and frailty. A previously unreported association with deprivation has been identified. The aetiology of deprivation is a multifactorial, affecting physical and mental health, often resulting in poor nutrition, long-term ill health and a short-end life span. That is known to affect health outcomes and the association with dysphagia should not be a surprise. Further research exploring the inter-relationship between dysphagia and health and social factors contributing to deprivation is indicated.

## Supplementary Information

Below is the link to the electronic supplementary material.

Supplementary file1 (DOCX 33 KB)
