# Supplementary material for: Identifying Dysphagia and Demographic Associations in Older Adults Using Electronic Health Records: A National Longitudinal Observational Study in Wales (United Kingdom) 2008–2018
Source: Dysphagia. 2022 Feb 25;37(6):1612–22. doi: 10.1007/s00455-022-10425-5 (PMC9643178; doi:10.1007/s00455-022-10425-5)
Supplement: Supplementary file 1 — Supplementary file1 (DOCX 33 KB) [file 455_2022_10425_MOESM1_ESM.docx]

Table S1. Distinct individuals aged 65+ with Welsh Index of Multiple Deprivation (WIMD) data in SAIL.

| **Year** | **2008** | **2009** | **2010** | **2011** | **2012** | **2013** | **2014** | **2015** | **2016** | **2017** | **2018** |
| --- | --- | --- | --- | --- | --- | --- | --- | --- | --- | --- | --- |
| **Cohort aged 65+** | 542,507 | 550,550 | 561,930 | 567,647 | 581,615 | 598,911 | 611,987 | 623,544 | 633,510 | 641,574 | 650,348 |
| **Cohort with General Practice data in SAIL** | 413,918 | 421,130 | 431,173 | 437,969 | 451,539 | 464,276 | 478,729 | 491,378 | 503,958 | 515,275 | 525,633 |
| **Cohort with WIMD data in SAIL** | 400,921 | 407,983 | 417,824 | 424,317 | 437,693 | 450,160 | 464,122 | 475,882 | 486,620 | 495,186 | 502,791 |

Table S2. Read code version 2 (CTV2) and ICD-10 code breakdowns for dysphagia diagnoses over time. Small counts of codes are masked as <10 due to governance requirements.

|  | **Cohort index date** | 2008 | 2009 | 2010 | 2011 | 2012 | 2013 | 2014 | 2015 | 2016 | 2017 | 2018 |
| --- | --- | --- | --- | --- | --- | --- | --- | --- | --- | --- | --- | --- |
|  | **Date range used** | 2007-2008 | 2008-2009 | 2009-2010 | 2010-2011 | 2011-2012 | 2012-2013 | 2013-2014 | 2014-2015 | 2015-2016 | 2016-2017 | 2017-2018 |
| **Read code (CTV2)** | **Read code description** |  |  |  |  |  |  |  |  |  |  |  |
| 194.. | Swallowing symptoms | 1178 | 1158 | 1205 | 1322 | 1353 | 1430 | 1439 | 1437 | 1527 | 1447 | 1419 |
| 1942. | Difficulty swallowing solids | 48 | 31 | 39 | 37 | 27 | 25 | 33 | 25 | 19 | 21 | 26 |
| 1943. | Difficulty swallowing liquids | <10 | <10 | <10 | <10 | 10 | <10 | <10 | <10 | <10 | <10 | <10 |
| 1944. | Painful swallowing | 30 | 34 | 20 | 30 | 34 | 34 | 45 | 43 | 45 | 30 | 38 |
| 1946. | Chokes when swallowing | - | - | - | - | - | - | - | - | <10 | <10 | <10 |
| 194Z. | Swallowing symptom NOS | 25 | 13 | 23 | 25 | 30 | 39 | 25 | 13 | 10 | <10 | 12 |
| 25W.. | Swallowing observation | - | - | - | - | - | - | - | <10 | - | 18 | 15 |
| 25W0. | O/E - swallowing normal | - | - | - | - | - | - | - | - | - | <10 | <10 |
| 25W1. | O/E - swallowing abnormality | - | - | - | - | - | - | - | - | - | - | <10 |
| 8T0K. | Referral to feeding and swallowing service | - | - | - | - | <10 | - | - | <10 | 34 | 39 | 65 |
| 9Oq7. | Dysphagia screening completed | - | - | - | <10 | <10 | - | <10 | <10 | - | <10 | <10 |
| D00y0 | Sideropenic dysphagia | <10 | <10 | - | - | <10 | <10 | - | - | - | - | - |
| R072. | Dysphagia | 125 | 127 | 112 | 118 | 104 | 107 | 117 | 82 | 72 | 91 | 68 |
| R0720 | Difficulty in swallowing | 255 | 276 | 253 | 294 | 327 | 346 | 343 | 366 | 459 | 399 | 422 |
| R072z | Dysphagia NOS | <10 | <10 | <10 | <10 | - | <10 | <10 | <10 | 12 | 14 | 15 |
| ZV416 | Problem with swallowing or mastication | 20 | 19 | 18 | 19 | 15 | 20 | 10 | <10 | <10 | <10 | <10 |
| **ICD-10 code** |  |  |  |  |  |  |  |  |  |  |  |  |
| R13 | Dysphagia | 1123 | 1119 | 1159 | 1216 | 1254 | 1399 | 1591 | 1713 | 2005 | 2057 | 2227 |

Table S3. Chi-squared p-values for the differences in proportions of age, frailty (eFI), gender and Welsh Index of Multiple Deprivation (WIMD) between individuals with and without a dysphagia diagnosis per year. p-values below the 0.05 significance level have been highlighted in bold font.

| Year | 2008 | 2009 | 2010 | 2011 | 2012 | 2013 | 2014 | 2015 | 2016 | 2017 | 2018 |
| --- | --- | --- | --- | --- | --- | --- | --- | --- | --- | --- | --- |
| Age | **<0.001** | **<0.001** | **<0.001** | **<0.001** | **<0.001** | **<0.001** | **<0.001** | **<0.001** | **<0.001** | **<0.001** | **<0.001** |
| eFI | **<0.001** | **<0.001** | **<0.001** | **<0.001** | **<0.001** | **<0.001** | **<0.001** | **<0.001** | **<0.001** | **<0.001** | **<0.001** |
| Gender | 0.390 | **0.003** | 0.621 | 0.133 | 0.174 | **0.049** | 0.342 | 0.053 | **0.030** | 0.650 | 0.126 |
| WIMD | **0.001** | **0.003** | **<0.001** | **0.006** | **<0.001** | **<0.001** | **<0.001** | **<0.001** | **<0.001** | **<0.001** | **<0.001** |

**Sensitivity analyses**

Multilevel logistic regression models were calculated for dysphagia diagnoses recorded in primary care (general practice) and secondary care (hospital) separately.

Table S4. Multilevel logistic regression models with dysphagia identified in primary care (general practice) records in the previous year as the outcome. WIMD: Welsh Index of Multiple Deprivation.

| **General Practice identified Dysphagia** | Null | Age | Gender | Frailty | WIMD | Multivariate |
| --- | --- | --- | --- | --- | --- | --- |
| Age (Reference: 65-74) |  |  |  |  |  |  |
| 75-84 |  | 1.521 (1.474,1.568) |  |  |  | 1.078 (1.044,1.114) |
| 85+ |  | 2.186 (2.107,2.268) |  |  |  | 1.200 (1.154,1.249) |
| Gender (Reference: Female) |  |  |  |  |  |  |
| Male |  |  | 0.968 (0.942,0.995) |  |  | 1.128 (1.097,1.160) |
| electronic Frailty Index (Reference: Fit) |  |  |  |  |  |  |
| Mild |  |  |  | 2.454 (2.365,2.547) |  | 2.412 (2.322,2.504) |
| Moderate |  |  |  | 4.731 (4.546,4.923) |  | 4.542 (4.354,4.737) |
| Severe |  |  |  | 8.436 (8.025,8.870) |  | 7.971 (7.558,8.406) |
| Welsh Index of Multiple Deprivation (Reference: 5.Least Deprived) |  |  |  |  |  |  |
| 1. Most deprived |  |  |  |  | 1.163 (1.113,1.214) | 1.008 (0.965,1.053) |
| 2 |  |  |  |  | 1.061 (1.017,1.107) | 0.957 (0.917,0.999) |
| 3 |  |  |  |  | 1.083 (1.039,1.129) | 1.001 (0.960,1.044) |
| 4 |  |  |  |  | 1.008 (0.965,1.052) | 0.958 (0.917,1.000) |
| Intercept | 0.004 (0.004,0.004) | 0.003 (0.003,0.003) | 0.004 (0.004,0.004) | 0.002 (0.002,0.002) | 0.004 (0.004,0.004) | 0.002 (0.002,0.002) |
|  |  |  |  |  |  |  |
| Random part estimate (Year) |  |  |  |  |  |  |
| Intercept variance | 0.00065 | 0.00077 | 0.00065 | 0.00037 | 0.00066 | 0.00036 |
| Standard error | 0.00051 | 0.00056 | 0.00051 | 0.00039 | 0.00051 | 0.00038 |

Table S5. Multilevel logistic regression models with dysphagia identified in secondary care (hospital) records in the previous year as the outcome. WIMD: Welsh Index of Multiple Deprivation.

| **Secondary care identified dysphagia** | Null | Age | Gender | Frailty | WIMD | Multivariate |
| --- | --- | --- | --- | --- | --- | --- |
| Age (Reference: 65-74) |  |  |  |  |  |  |
| 75-84 |  | 1.570 (1.517,1.625) |  |  |  | 1.113 (1.074,1.153) |
| 85+ |  | 2.235 (2.145,2.327) |  |  |  | 1.236 (1.183,1.291) |
| Gender (Reference: Female) |  |  |  |  |  |  |
| Male |  |  | 1.012 (0.982,1.044) |  |  | 1.185 (1.149,1.222) |
| electronic Frailty Index (Reference: Fit) |  |  |  |  |  |  |
| Mild |  |  |  | 2.571 (2.466,2.680) |  | 2.499 (2.396,2.607) |
| Moderate |  |  |  | 5.070 (4.849,5.300) |  | 4.775 (4.556,5.004) |
| Severe |  |  |  | 8.458 (7.993,8.950) |  | 7.819 (7.365,8.302) |
| Welsh Index of Multiple Deprivation (Reference: 5.Least Deprived) |  |  |  |  |  |  |
| 1. Most deprived |  |  |  |  | 1.413 (1.348,1.482) | 1.223 (1.166,1.282) |
| 2 |  |  |  |  | 1.201 (1.145,1.258) | 1.080 (1.030,1.132) |
| 3 |  |  |  |  | 1.020 (0.971,1.070) | 0.941 (0.896,0.987) |
| 4 |  |  |  |  | 1.015 (0.966,1.066) | 0.963 (0.917,1.012) |
| Intercept | 0.003 (0.003,0.004) | 0.003 (0.002,0.003) | 0.003 (0.003,0.004) | 0.001 (0.001,0.002) | 0.003 (0.003,0.003) | 0.001 (0.001,0.001) |
| Random part estimate (Year) |  |  |  |  |  |  |
| Intercept variance | 0.03194 | 0.03385 | 0.03191 | 0.03548 | 0.03253 | 0.03542 |
| Standard error | 0.01389 | 0.01469 | 0.01388 | 0.01529 | 0.01414 | 0.01527 |
